# Syndrome de cri du chat révélé par une dysmorphie cranio-faciale et faible succion chez un nouveau-né: à propos d’un cas

**DOI:** 10.11604/pamj.2023.46.109.42239

**Published:** 2023-12-19

**Authors:** Ahmed Hared Bouh, Mouad Nejjari, Abdisalam Oumar Hassan, Nouzha Dini, Inssaf AL Ammari

**Affiliations:** 1 Département de Pédiatrie-Néonatologie, Hôpital Universitaire International Mohammed VI, Université Mohammed VI des Sciences et de la Santé, Casablanca, Maroc; 2 Faculté de Médecine et Pharmacie, Université Mohammed V, Rabat, Maroc

**Keywords:** Dysmorphie cranio-faciale, nouveau-né, faible succion, cri du chat, cas clinique, Facial dysmorphism, newborn, weak suction, cri-du-chat syndrome, case report

## Abstract

Le syndrome du cri du chat est une maladie génétique rare, due à une délétion du bras court du chromosome 5 (5p-) avec une incidence comprise entre 1/15000 et 1/50000 nouveau-nés vivants. Il s'agit d'un nouveau-né de sexe masculin âgé d'un jour, issu d'un mariage non consanguin et de mère primigeste, la grossesse s'est déroulée sans complications et menée à terme. L´accouchement était effectué par césarienne en urgence devant une suspicion d´une chorio-amniotite avec bonne adaptation à la vie extra-utérine et un poids de naissance à 2295g. L'examen clinique a révélé une dysmorphie crânio-faciale avec hypertélorisme et microcéphalie, une hypotonie, une mauvaise succion et un pied bot plus marqué à droite, le reste de l'examen étant sans particularité. Pendant l'hospitalisation, un cri aigu monochromatique imitant le miaulement d'un chat a été observé. Le diagnostic clinique a été confirmé par la fluorescence in situ hybridization, qui a montré une délétion du bras court du chromosome 5 (5p15.2). Le bilan malformatif de base n'a révélé aucune autre anomalie. L'association d'un cri aigu monochromatique et d'une dysmorphie crânio-faciale chez un nouveau-né doit faire évoquer la nécessité d'une étude cytogénétique, en particulier d'une hybridation in situ en fluorescence

## Abstract (en)

Cri-du-chat syndrome is a rare genetic disorder, due to a deletion of the short arm of chromosome 5 (5p-). Its incidence is ranging from 1/15000 to 1/50000 live births. This was a one-day-old male newborn from a non-consanguineous marriage, the first pregnancy uncomplicated and carried to term with a birth weight of 2295g. Clinical examination revealed: craniofacial dysmorphism with hypertelorism and microcephaly, hypotonia, poor suction and clubfoot more marked on the right, the rest of the examination was unremarkable. During hospitalization, a high-pitched monochromatic cry mimicking a cat's meow was observed. The clinical diagnosis was confirmed by fluorescence in situ hybridization, showing a deletion of the short arm of chromosome 5 (5p15.2). The basic malformative work-up came back without any other abnormalities. The association of a high-pitched monochromatic cry with craniofacial dysmorphism in a newborn should indicate the need for cytogenetic study, in particular fluorescence in siti hybridization

## Introduction

Le syndrome du cri-du-chat est une maladie génétique rare due à une délétion du bras court du chromosome 5 (5p-) [1]. Son incidence varie de 1/15 000 à 1/50 000 des nouveau-nés vivants. La principale caractéristique de ce syndrome en période néonatale est un cri aigu monochromatique semblable à celui d'un chat [2]. Décrit pour la 1^ère^ fois en 1963 par Lejeune le syndrome clinique est confirmé par l´étude cytogénétique, notamment la fluorescence in situ hybridization (FISH) qui dispose de sonde spécifique pour la maladie du cri du chat. Le traitement est symptomatique. Nous présentons l´observation d´un syndrome de cri du chat chez un nouveau-né confirmé par l´exploration cytogénétique. A travers cette observation, nous décrivons les profils clinique, cytogénétique et pronostique de cette maladie rare

## Patient et observation

**Présentation du patient:** nouveau-né à J1 de vie, sexe masculin ,issu d´un mariage non consanguin et d´une 1^ère^ grossesse non compliquée et menée à terme, absence d´antécédents familiaux, des maladies génétiques, né par césarienne devant une suspicion de chorio-amniotite. L´adaptation à la vie extra-utérine était bonne avec un score d´APGAR à 8/10 et poids de naissance: 2295g, taille de naissance: 48cm, périmètre crânien: 31cm (-3DS), dysmorphie faciale et pieds bots plus accentués à droite.

**Examen clinique:** il était admis à J1 de vie pour dysmorphie cranio-faciale et refus de tétée avec examen à l´admission: SaO_2_: 95% à l´air ambiant, FC: 142 bat/min, TRC< 3s, bébé rose, dysmorphie cranio-faciale notamment microcranie et hypertélorisme, hypotonie, fontanelle normo tendue, faible succion, eupneique, auscultation cardiopulmonaire sans anomalies, abdomen souple et dépressible, pas de viscéromegalie, pieds bot plus marqué à droite, reste de l´examen était sans particularités.

**Figure 1:**
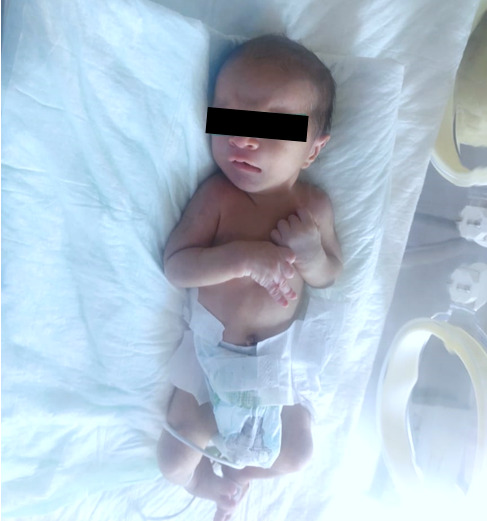
photo du nouveau-né montrant une dysmorphie faciale et pied bot plus marqué à droite

**Démarche diagnostique:** au cours de l´hospitalisation, il a été constaté un cri aigu monochromatique mimant un chat qui miaule. La Figure 1 illustre la dysmorphie cranio-faciale ainsi que le pied bot plus prononcé à droite. Le Tableau 1 montre les résultats de l´analyse biologique standard et sérologique. Une radiographie thoracique avec sonde nasogastrique en place a été faite et revenue sans anomalie. Une étude cytogénétique constitutionnelle (FISH) a été réalisée: l'analyse par les techniques de cytogénétique moléculaire à partir des sondes spécifiques du cri du chat (sonde Vsys) a mis en évidence une micro délétion du locus en 5p15.2 dans toutes les cellules inter phasiques étudiées. Ce résultat est en corrélation avec la suspicion diagnostique.

**Tableau 1:** résultats d’analyse biologique standard et sérologique

Noms de bilans	Valeurs	Valeurs de référence
Globules blancs	17.64 x 103/mm^3^	7- 34 x 103
Hémoglobine	17.3 g/dl	14.5- 22.5
Plaquettes	271 x 103/mm^3^	210-500
C Reactiv protein	1.6 mg/l	0.1- 4.1
Sodium	141mmol/l	136-145
Potassium	3.8mmol/l	3.8- 5.1
Chlorure	110 mmol/l	98-107
Réserves alcalines	15mmol/l	22- 29
Protidémie	65g/l	44-76
Calcium	88mg/l	76-104
Sérologie herpès IgG	5.4 UI/L	< 0.8
Sérologie herpès IgM	0.1 UI/L	< 0.9
Sérologie toxoplasmique IgM	0.04 UI/mL	<0.6
Sérologie toxoplasmique IgG	0 UI/mL	<3
Sérologie rubéolique IgM	0.02UI/mL	<1.2
Sérologie rubéolique IgG	8UI/mL	<5
Sérologie syphilis	0.08UI/mL	<1.00
Sérologie CMV IgM/IgG	négative	<0.8UI/mL
Hémoculture	Stérile	

Bilan infectieux négatif, sérologies toxoplasmose et cmv négatives, rubéole et herpès: profils immunisés

**Interventions thérapeutiques:** devant l´anamnèse infectieuse positive (suspicion chorio-amniotite et faible succion), le nouveau-né a été mis initialement sous une antibiothérapie probabiliste (cefotaxim 100mg/kg/12h IV et gentamycine 3mg/kg/j IVL, alimentation parentérale puis entérale par gavage et soins du nouveau-né.

**Suivi et résultats des interventions thérapeutiques:** un bilan malformatif de principe a été pratiqué et revenu sans anomalies (échographies transfontanellaire, cardiaque et abdominale, IRM cérébrale). Un examen ophtalmologique spécialisé était sans particularités. Les parents ont été informés sur la maladie, son pronostic à long terme et l´intérêt d´un conseil génétique lors d´une éventuelle grossesse ultérieure. Des séances d´éducation thérapeutique ont été données aux parents. L´évolution clinique était marquée par la stabilité respiratoire et hémodynamique. L´antibiothérapie était arrêtée après la négativité de bilan infectieux; il a été mis sortant à J15 de vie.

**Consentement**: le consentement éclairé de deux parents a été obtenu pour la publication et utilisation de la photo.

## Discussion

C´est en 1963 que Lejeune a décrit pour la 1^ère^ fois le syndrome de cri du chat. Il est dû à une délétion du bras court du chromosome 5. Il s´agit d´une maladie génétique rare avec une incidence allant de 1/15000 à 1/50000 nouveau-nés vivants. La mutation responsable de la délétion est de novo dans 85% des cas selon la revue italienne de Maria Elena Liverani *et al*. [3]. La principale caractéristique clinique est le cri aigu monochromatique mimant un cri de chat notamment aux premiers mois [1-3]. Notre patient admis à J1 de vie présentait à l´examen: la dysmorphie cranio-faciale (microcéphalie, hypertélorisme), faible succion, hypotonie, faible poids, pied bot et cri du chat. Ces signes cliniques constituent les principales caractéristiques cliniques en période néonatale. Le cri aigu, monotone et faible mimant un cri de chat est typique chez les nouveau-nés. Ce cri est en rapport avec une altération morphologique ou fonctionnelle du larynx, et disparait au cours des premiers mois ou années. La faible succion entraine une difficulté d´apport alimentaire et ou parfois des complications (reflux gastro-œsophagien, infections respiratoires récurrentes) [3-4]. L´association de la maladie de cri du chat à d´autres malformations cardiaques, neurologiques et autres n´est pas rare. Les patients vivants avec la maladie de cri du chat présentent des anomalies cardiaques dans 15% à 20% des cas. Le pronostic de cette maladie n´est pas satisfaisant. La délétion 5p induit un retard de langage et neurodéveloppemental constaté durant le suivi au long cours [3,5]. Dans notre cas, le bilan malformatif de principe (échographies cardiaque, abdominale et transfontanellaire, IRM Cérébrale) n´a décelé aucune anomalie associée. Le cri du chat est le principal signe clinique spécifique de la maladie d´où son appellation. Il doit conduire à la réalisation de l´analyse cytogénétique « FISH » même en absence de trait dysmorphique. La technique de la FISH permet d´appliquer une sonde spécifique (vsys) au CDCs et a amélioré la performance diagnostique par rapport à la cytogénétique conventionnelle. Elle permet de détecter des délétions de petite taille même inférieure à 5mb. Dans notre cas, la réalisation de la FISH a permis d´identifier la délétion 5p15.2, ce qui a confirmé le diagnostic. La sévérité des symptômes dépend de la taille de la délétion chromosomique [5,6]. Le syndrome de cri est une maladie complexe et handicapante. Il s´agit d´un fardeau pour la famille mais aussi pour la société. Selon l´étude de recherche par Kodra *et al*. Les patients vivants avec la maladie de cri du chat sont essentiellement tiers-dépendants, la charge de la pathologie est lourde pour les familles, ainsi elle engendre un impact économique et social d´où la nécessité d´élaborer des stratégies de santé publique ciblant cette maladie rare [7]. L´association de syndrome de cri du chat à d´autres anomalies notamment neurologique (myelomeningocèle, malformation du cervelet et du tronc cérébral aggrave le pronostic d´avantage. La recherche des gènes spécifiques à ces associations serait utile pour le diagnostic et le pronostic selon l´étude de Fatimah A Alabbad *et al*. [8].

## Conclusion

Le cri du chat et la dysmorphie cranio-faciale sont les principales caractéristiques cliniques de cette maladie durant la période néonatale. La présence de ces signes cliniques doit indiquer la réalisation de l´analyse cytogénétique notamment la Fish. Notre patient présentait le syndrome de cri du chat typique (cri aigu monochromatique, dysmorphie cranio-faciale, hypotonie, faible succion et faible poids, pied bot). Cette maladie induit un retard mental, de langage et psychomoteur, d´où la nécessité d´un conseil génétique lors d´une grossesse ultérieure. La prise en charge est multidisciplinaire et constitue un fardeau social.
